# Dexmedetomidine protects against sepsis-induced lung injury through autophagy and Smad2/3 signaling pathway

**DOI:** 10.22038/IJBMS.2023.73479.15964

**Published:** 2024

**Authors:** Zhanli Liu, Jiqing Xu, Yanqiu Zhao, Yanbin Wan, Rui Guo, Canling Long, Jia Liu, Xinhuang Yao, Wenchao Yin

**Affiliations:** 1 Department of Anesthesiology, Shenzhen People’s Hospital and Shenzhen Anesthesiology Engineering Center, the Second Clinical Medical College of Jinan University, Shenzhen, China; 2 Department of Cardiothoracic Surgery, The Second Affiliated Hospital, School of Medicine, The Chinese University of Hong Kong, Shenzhen & Longgang District People’s Hospital of Shenzhen, Shenzhen, China; 3 Central Laboratory, The Second Affiliated Hospital, School of Medicine, The Chinese University of Hong Kong, Shenzhen & Longgang District People’s Hospital of Shenzhen, Shenzhen, China; 4 Department of Anesthesiology, Sichuan Provincial Orthopedic Hospital, Chengdu, China

**Keywords:** Acute lung injury, Autophagy, Dexmedetomidine, Sepsis, Smad2/3

## Abstract

**Objective(s):**

Dexmedetomidine (Dex) is a potent α2-adrenergic receptor(α2-AR) agonist that has been shown to protect against sepsis-induced lung injury, however, the underlying mechanisms of this protection are not fully understood. Autophagy and the Smad2/3 signaling pathway play important roles in sepsis-induced lung injury, but the relationship between Dex and Smad2/3 is not clear. This study aimed to investigate the role of autophagy and the Smad2/3 signaling pathway in Dex-mediated treatment of sepsis-induced lung injury. Sepsis was performed using cecal ligation and puncture (CLP) in C57BL/6J mice.

**Materials and Methods:**

Mice were randomly assigned to four groups (n=6 per group): sham, CLP, CLP-Dex, and CLP-Dex-YOH, Yohimbine hydrochloride (YOH) is an α2-AR blocker. The cecum was carefully separated to avoid blood vessel damage and was identified and punctured twice with an 18-gauge needle. The pathological changes, inflammatory factor levels, oxidative stress, autophagy, Smad2/3 signaling pathway-related protein levels in lung tissues, and the activity of superoxide dismutase (SOD) and malonaldehyde (MDA) in the serum were measured.

**Results:**

CLP-induced lung injury was reflected by increased levels of inflammatory cytokines, apoptosis, and oxidative stress, along with an increase in the expression of autophagy and Smad2/3 signaling pathway-related proteins. Dex could reverse these changes and confer a protective effect on the lung during sepsis. However, the administration of YOH significantly reduced the positive effects of Dex in mice with sepsis.

**Conclusion:**

Dex exerts its beneficial effects against sepsis-induced lung injury through the regulation of autophagy and the Smad2/3 signaling pathway.

## Introduction

Sepsis, a systemic inflammatory response to significant infection, remains a leading cause of death worldwide. Acute lung injury (ALI) is a hallmark of sepsis, characterized by apoptosis of the pulmonary epithelial cells, driven by the activation of several inflammatory signaling mediators in the lung. These inflammatory mediators release proinflammatory cytokines, including tumor necrosis factor (TNF) and interleukins (ILs) such as IL-1β and IL-6, ultimately triggering a cascade of inflammation and apoptosis in pulmonary epithelial cells (1). 

Dexmedetomidine (Dex), a highly selective α2-adrenergic receptor (α2-AR) agonist, possesses significant anti-inflammatory and anti-oxidative stress properties. Consequently, Dex has found extensive use in the treatment of conditions like sepsis, myocardial ischemia-reperfusion injury, and brain injury (2). Conversely, YOH, an α2-AR inhibitor, is frequently employed in research as a Dex antagonist. Recent clinical experiments have shown that Dex can reduce inflammatory factors in sepsis patients (3). However, the precise mechanism underlying this effect has not been fully elucidated. 

Autophagy is a lysosome-dependent degradation process crucial for maintaining cellular homeostasis. It operates as a protective mechanism to recycle these damaged components into energy, thereby maintaining intracellular homeostasis, especially during stressful conditions. Previous research on ALI has illuminated the intricate relationship between local inflammatory responses and heightened autophagic activity, signifying the adaptive role of autophagy in the initial phases of sepsis-induced inflammation (4). Apoptosis is a highly conserved process from *Caenorhabditis elegans* to humans. It plays vital roles in vertebrates during development, cellular homeostasis, and tumor suppression. apoptosis is characterized by a signaling cascade that activates executioner caspases, leading to the efficient clearance of intracellular components and eventual phagocytosis of the dying cell. Autophagy has closed connections between cell death and apoptosis, in some cases, the same proteins control both autophagy and apoptosis such as Beclin1 and Bcl-2, apoptosis signaling and autophagy can be mutually regulated (5). However, distinguishing between apoptotic and autophagic cell death is challenging, as these two processes are not mutually exclusive; instead, the type and extent of stress determine whether a cell’s response shifts from adaptive autophagy to apoptotic cell death. Beclin-1, recognized as an autophagic marker, plays a pivotal role in orchestrating the localization of other autophagic proteins to the autophagosome prestructure (6). The mammalian target of rapamycin (mTOR), another key factor in autophagy, is widely regarded as the principal regulator of this process (7), these two proteins are the main markers of autophagy. In sepsis, the activation of autophagy can help alleviate excessive cytokine release and mitigate sepsis-induced pulmonary epithelial cell apoptosis (1). Notably, sepsis also leads to an increase in the number of damaged mitochondria, which can be selectively targeted and eliminated through autophagy (8). Autophagy dysregulation has been shown to occur in various lung diseases, including lung damage (9, 10), lung fibrosis (11), and chronic obstructive pulmonary disease (12). Furthermore, autophagy has been linked to multiple organ damage and dysfunction resulting from sepsis (13). This evidence underscores the contribution of autophagy to the development of sepsis and sepsis-induced lung injury.

The transforming growth factor-β1 (TGF-β1)/Smad2/3 signaling pathway has been implicated in modulating the inflammatory response in conditions such as pulmonary fibrosis following Pirfenidone administration (14). Quercetin has been shown to ameliorate kidney injury by modulating macrophage polarization, reducing excessive extracellular matrix accumulation and interstitial fibrosis through its antagonistic action on the TGF-β1/Smad2/3 signaling pathway (15). Notably, the TGF-β1/Smad2/3 signaling pathway also plays a pivotal role in maintaining the equilibrium between proinflammatory and anti-inflammatory cytokines (16), underscoring the essential role in immune responses. 

We postulate that the protective effects of Dex are intricately linked to autophagy and the Smad2/3 signaling pathway. Therefore, this study was designed to investigate how Dex administration in mice can safeguard against cecal ligation and puncture (CLP)-induced lung injury and mortality by modulating autophagy and the Smad2/3 signaling pathway.

## Materials and Methods

***CLP Model***

Male C57BL/6 mice were sourced from the Guangdong Medical Lab Animal Center and housed at the Laboratory Animal Service Center (Jinan University, Jinan, China). According to the Animal Care Guidelines of Jinan University, animals received standard care under a 12 hr dark/light cycle and were given free access to food and water. Sepsis was induced using CLP, as previously described (17). The mice were anesthetized using pentobarbital sodium according to standard protocol. The cecum was carefully separated to avoid blood vessel damage and was identified and punctured twice with an 18-gauge needle. Before the cecum was returned to the abdominal cavity, feces were gently extruded. The abdominal cavity was quickly closed with two epithelium layers, followed by subcutaneous fluid injection to wake the mice before returning to the cage. An overdose of phenobarbital sodium was used for euthanasia to allow collection of blood and lung samples.

***Animal experimental protocol***

Mice were randomly divided into four groups (n=6 per group): Sham, CLP, CLP+Dex, and CLP+Dex+YOH, YOH is an α2-AR. Based on our preliminary experiments and previous reports (17-19), Dex was administered 15 min before inducing sepsis at a dose of 50 μg/kg, with YOH added at a dose of 1 mg/kg 30 min before Dex administration. After 24 hr of sepsis, blood samples were collected for serum separation, and the lungs were harvested and weighed. Tibial lengths were also measured. Dex was sourced from Jiangsu Hengrui Medicine Co., Ltd. (Lianyungang, China), and YOH was obtained from MedChem Express (Monmouth Junction, NJ, USA).

***Hematoxylin and eosin (H*** ***&*** ***E) staining***

Lung tissues were fixed with 4% paraformaldehyde overnight at room temperature, embedded in paraffin, and sliced into 4 μmthick slices. These tissues were stained using an H&E staining kit (catalog number: C0105; Beyotime Institute of Biotechnology). Slides were visualized under a fluorescence microscope (DMi8, DFC7000 T; Leica Microsystems, Inc.).

***Determination of IL-1β, TNF-α, MDA, and SOD release***

Blood samples collected were subjected to centrifugation to isolate serum, enabling the measurement of IL-1β, TNF-α, MDA, and SOD levels. Corresponding ELISA kits were obtained from Shanghai Jianglai Biological Technology Co., Ltd. (Shanghai, China) and assays were conducted as per the manufacturer’s instructions.

***Reverse transcription-quantitative polymerase chain reaction (RT-qPCR)***

Total RNA was isolated using Tirol reagent (Invitrogen, catalog number: 15596026), according to the manufacturer’s protocol. Subsequently, reverse transcription reactions and real-time PCR were performed according to the manufacturer’s protocol. The reactions were performed using the PrimeScript RT reagent kit (Takara, catalog number: RR037A). RT-qPCR was performed with a final volume of 20 μl (SYBR® Green Premix Pro Taq HS qPCR Kit, catalog number: AG11718; Accurate Biology). GAPDH was used as an internal control to normalize the mRNA levels of gene expression. The primer sequences used were as follows: GAPDH, F 5’-TCCTGCACCACCAACTGCTTAG-3’, R 5’-GTCAGATCCACGACGGACACAT-3’; IL-1β F 5’-CTCGCAGCAGCACATCAAC-3’, R 5’-GTTCATCTCGGAGCC-TGTAGT-3’; IL-6, F 5’-GGAGAGGAGACTTCACAGAGG-3’, R 5’-CCAGTTT-GGTAGCATCCATCAT-3’; TNF, F 5’-GGAACTGGCAGAAGAGGCACTC-3’, R 5’-CCATAGAACTGATGAGAGGGAGGC-3’. All reactions, including no-template controls, were run in triplicate using QuantStudio™ 3 (Applied Biosystems).

***Western blot analysis***

Frozen lung tissue samples were homogenized in RIPA buffer (Cell Signaling Technology, Inc., catalog number: 9806; Inc., Danvers, MA, USA) and centrifuged at 13,200 ×g for 30 min. The resulting supernatant was used for total protein analysis, and the protein concentration was measured using the Bradford protein assay. Equal amounts of protein samples were loaded and run on 7.5-12.5% SDS-PAGE and subsequently transferred to PVDF membranes (Millipore). The membranes were then blocked in TBST containing 5% non-fat milk for 1 hr at room temperature, after which the membranes were incubated with primary antibodies overnight at 4 ^°^C. Primary antibodies Bax (catalog number: 2772; Cell Signaling Technology, Inc.), B-cell lymphoma 2 (Bcl-2)(catalog number: 210774; Merck KGaA, Darmstadt, Germany), cleaved caspase-3 (catalog number: 836; Cell Signaling Technology, Inc.), Smad2/3 (catalog number: 3102s; Cell Signaling Technology, Inc.), p-Smad2/3 (catalog number: 8828s; Cell Signaling Technology, Inc.), mTOR (catalog number: 2983s; Cell Signaling Technology, Inc.), p-mTOR (catalog number: 5536s; Cell Signaling Technology, Inc.), and GAPDH (catalog number: 5174; Cell Signaling Technology, Inc. MA, USA) were used at a dilution of 1:1000. After washing three times for 10 min each, the membrane strips were incubated for 1 hr with a 1:10 000 dilution of an anti-rabbit or anti-mouse IgG secondary antibody (Cell Signaling Technology, Inc.) conjugated to horseradish peroxidase. Protein bands were detected using enhanced chemiluminescence, and the images were quantified using a densitometer.

***Statistical analysis***

All values are expressed as mean±standard error of the mean (±SEM). Statistical analyses were performed using GraphPad Prism 8 (GraphPad Software, San Diego, CA, USA). All data were normally distributed, as confirmed by normality tests. Differences among multiple groups were analyzed using one-way ANOVA, followed by Tukey’s test for multiple comparisons. *P*<0.05 was deemed statistically significant.

## Results

***Characterization of sepsis-induced lung injury***

**Figure 1:**
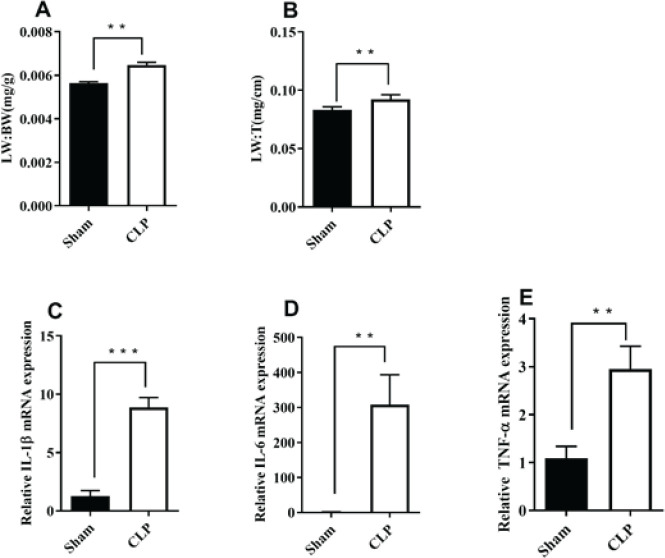
Acute lung injury (ALI) induced by cecal ligation and puncture (CLP)

Sepsis-induced ALI was induced through CLP in mice. The results displayed in Figure 1A-B indicate that the lung weight/body weight (LW/BW) ratio and lung weight/tibia length (LW/TL) ratio significantly increased following CLP in mice (**P**<**0.01, CLP+PBS vs Sham). Furthermore, the levels of inflammatory factors, including IL-1β, IL-6, and TNF-α, were markedly elevated after CLP (**P**<**0.01, CLP+PBS vs Sham; Figure 1C-E). These findings suggest that CLP effectively induced ALI, and inflammation played a crucial role in this process.

***Effects of Dex on lung pathological injury and inflammation level in sepsis-induced lung injury***

**Figure 2:**
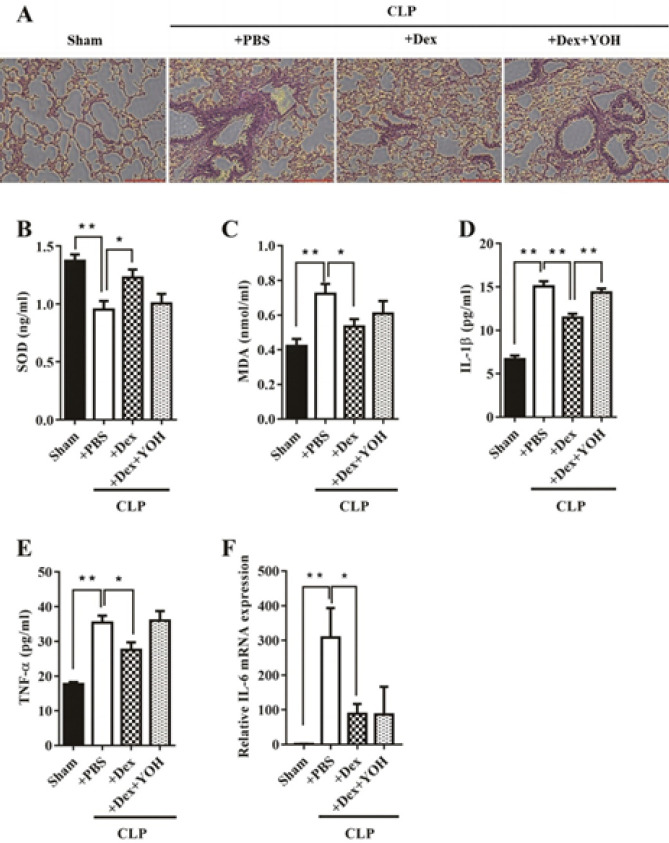
Dex attenuates inflammation and oxidative stress in CLP-induced ALI

Examination of lung tissues from the four groups using H&E staining revealed distinct findings, under physiological conditions, lung tissues exhibited thin alveolar walls, clear alveolar spaces without exudation, and a lack of inflammatory cell infiltration (Figure 2A). However, in septic mice (CLP+PBS group), lung tissue displayed significant pathological changes, including diffuse neutrophils, hyaline membrane formation, diffuse thickening of alveolar walls, and alveolar wall disruption. Dex treatment exacerbated inflammatory cell infiltration and disrupted alveolar structures in septic lung tissues. Intriguingly, the addition of YOH in the CLP+Dex+YOH group intensified the pathological changes observed in septic lung tissues.

The CLP procedure led to a significant decrease in the serum antioxidant SOD level and an increase in MDA levels, contributing to lung injury during CLP (**P**<**0.01, CLP+PBS vs Sham). Dex treatment (CLP+Dex group) significantly enhanced SOD release and reduced MDA release compared to the CLP+PBS group (*P**<**0.05, CLP+Dex vs CLP+PBS). However, the addition of YOH reversed the positive effects of Dex. CLP substantially increased the levels of inflammatory cytokines, including IL-1β and TNF-α in serum and IL-6 in lung tissues, when compared to the sham group (all **P**<**0.01, CLP+PBS vs sham). Dex significantly lowered the levels of all three cytokines induced by CLP (*P**<**0.05, CLP+Dex vs CLP+PBS), while YOH counteracted the protective effects of Dex (*P**<**0.05, CLP+Dex+YOH vs CLP+Dex). These data indicate that Dex reduced lung damage and inflammation induced by CLP, but YOH nullified the protective effects of Dex.

***Effects of Dex on apoptosis in pulmonary damage of sepsis mouse model***

**Figure 3.**
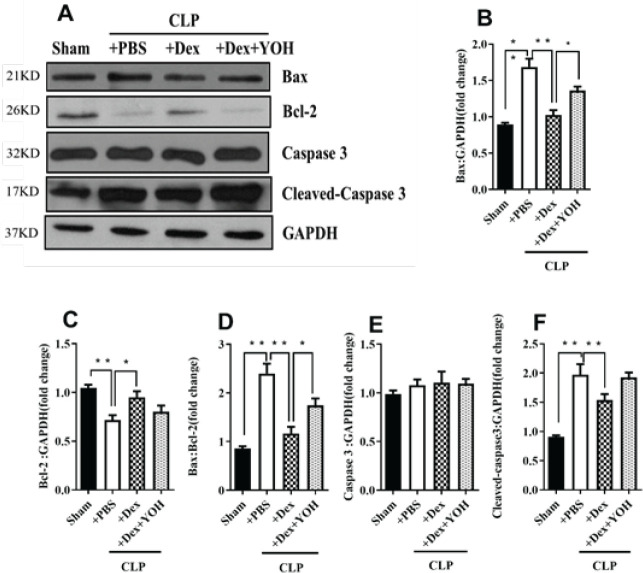
Dex attenuates apoptosis in CLP-induced ALI

Apoptosis is associated with ALI. Thus, we assessed the levels of apoptosis in pulmonary damage induced by sepsis. As demonstrated in Figure 3A-C, the expression of the apoptosis-associated protein Bax was significantly increased, whereas the anti-apoptotic protein Bcl-2 was substantially down-regulated after CLP (**P**<**0.01, CLP+PBS vs Sham). Dex treatment reversed these changes and reduced ALI (*P**<**0.05, CLP+Dex vs CLP+PBS). However, the addition of YOH counteracted the effects of Dex (*P<0.05, CLP+Dex+YOH vs CLP+Dex). Moreover, caspase-3 and cleaved caspase-3 expressions were markedly increased in the lung tissue of the sepsis group. Dex treatment similarly mitigated these effects. Interestingly, the effects of Dex on caspase-3 and cleaved caspase-3 proteins were not relieved by supplementation with YOH (Figures 3A, E, and F).

***Effects of Dex on autophagy-related signaling in sepsis-induced lung injury***

**Figure 4:**
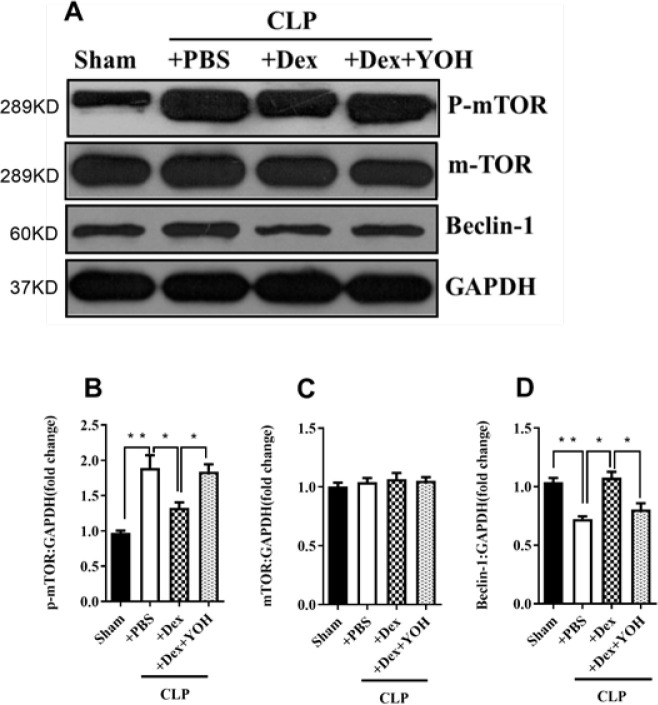
Effects of Dex on the expression of autophagy-related protein in CLP-induced ALI

Among the autophagy-related indicators, CLP up-regulated phosphorylated mTOR (p-mTOR) and down-regulated Beclin-1 expression in lung tissue. However, Dex treatment reversed these changes (p-mTOR, **P**<**0.01, CLP+PBS vs Sham; Beclin-1, **P**<**0.01, CLP+PBS vs Sham; Figure 4A-C). Importantly, the addition of YOH counteracted the effects of Dex on the expression of these autophagy-related proteins (p-mTOR, *P**<**0.05, CLP+PBS vs Sham; Beclin-1, *P**<**0.05, CLP+PBS vs Sham; Figure 4A-C).

***Effects of Dex on Smad2/3-related signaling in sepsis-induced lung injury***

**Figure 5:**
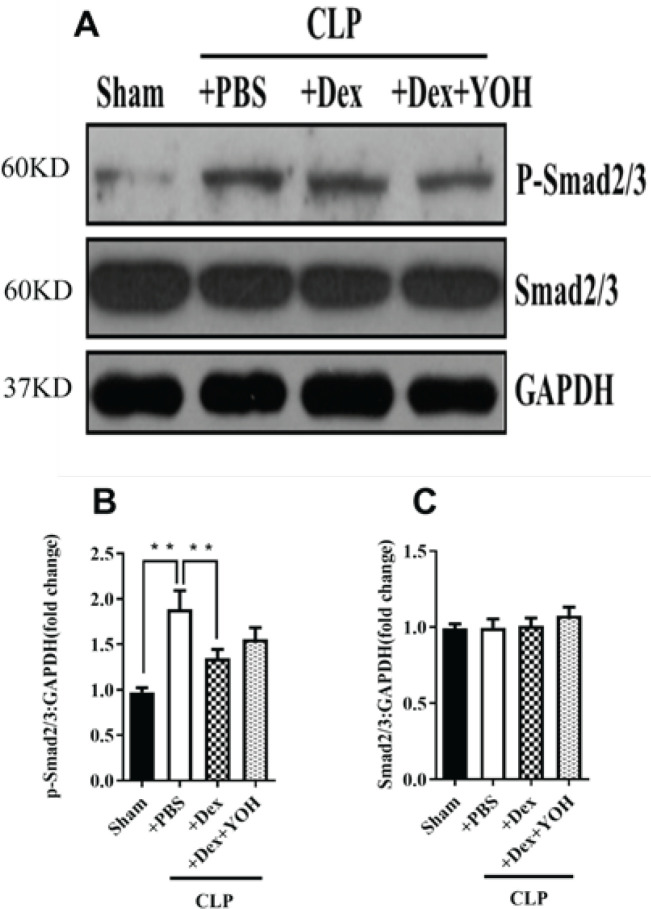
Effects of Dex on the expression of Smad2/3-related protein in CLP-induced ALI

As illustrated in Figure 5, the expression of phosphorylated Smad2/3 (p-Smad2/3), but not total Smad2/3, was elevated in the lung tissue of septic mice and could be restored by Dex treatment (**P**<**0.01, CLP+PBS vs Sham; **P**<**0.01, CLP+DEX vs CLP+PBS). In contrast, YOH slightly attenuated the effects of Dex and increased the protein levels of p-Smad2/3 in the lung tissue of septic mice (Figure 5).

A. Ratio of Lung/Body weight; B. Ratio of Lung weight/Tibia length; C-E. Levels of IL-1β (C), IL-6 (D), and TNF-α (E) gene expression in the lungs after cecal ligation and puncture (CLP) operation were measured by real-time PCR. Sepsis was achieved via CLP

Data are presented as the mean±SEM. n=6 per group. **P<0.01 and ***P<0.001. Sham, control; ns, not significant

A) Role of Dex in pathological lung injury in septic mouse models for Sham, CLP+PBS, CLP+Dex, and CLP+Dex+YOH groups. B-C) Changes in SOD (B) and MDA (C) release in the serum determined using ELISA. D-F) Content of inflammatory cytokines in lung tissues, including IL-1β (D) and TNF-α (E) in serum and IL-6 (F). Sepsis was achieved via CLP. Data are presented as the mean±SEM. n=6 per group. *P<0.05 and **P<0.01

Dex: dexmedetomidine; CLP: cecal ligation and puncture; Sham, control; PBS: Phosphate buffered saline; YOH: yohimbine hydrochloride; SOD: superoxide dismutase; MDA: malonaldehyde; ALI: acute lung injury

A) Western blotting analysis of apoptosis-related protein expression in lung tissues of sepsis mice. B-D) Quantitative changes in the protein expression of Bax (B), Bcl-2 (C), caspase-3 (E), and cleaved caspase-3 (F) in lung tissues for Sham, CLP+PBS, CLP+Dex, and CLP+Dex+YOH groups

Data are presented as the mean±SEM. n=6 per group. *P<0.05 and **P<0.01

Dex: dexmedetomidine; Sham, control; CLP: cecal ligation and puncture; PBS: Phosphate buffered saline; YOH: yohimbine hydrochloride

A) Analysis by western blotting of autophagy-related protein expression in lung tissues of sepsis mice. B-F) Quantitative changes in the protein expression of p-mTOR (B), mTOR (C), and Beclin-1 (D) in lung tissues for Sham, CLP+PBS, CLP+Dex, and CLP+Dex+YOH groups

Data are presented as the mean±SEM. n=6 per group. *P<0.05 and **P<0.01

Dex: dexmedetomidine; Sham, control; CLP: cecal ligation and puncture; PBS: Phosphate buffered saline; YOH: yohimbine hydrochloride

A) Western blotting analysis of Smad2/3-related protein expression in lung tissues of sepsis mice. B-C) Quantitative changes in the protein expression of p-Smad2/3 (B) and Smad2/3 (C) in lung tissues for Sham, CLP+PBS, CLP+Dex, and CLP+Dex+YOH groups

Data are presented as the mean±SEM. n=6 per group. **P<0.01

Dex: dexmedetomidine; Sham, control; CLP: cecal ligation and puncture; PBS: Phosphate buffered saline; YOH: yohimbine hydrochloride

## Discussion

Sepsis continues to pose a formidable challenge in clinical practice, while ALI remains a life-threatening condition with a significant global burden of morbidity and mortality. Central to the pathophysiology of sepsis is a pronounced inflammatory response. This cascade of inflammation not only precipitates oxidative stress but also leads to lipid peroxidation, DNA damage, and impairment of mitochondrial function. Reciprocally, oxidative stress can initiate and perpetuate inflammation, mediated by redox reactions involving circulating inflammatory factors and the nuclear factor kappa-light-chain-enhancer of activated B-cells (NF-κB) (20). In our study, we found that inflammatory factors and oxidative stress were significantly increased, this demonstrated that they played important roles in ALI.

Moreover, autophagy serves as a key regulator of antioxidant and anti-inflammatory homeostasis. Over the past few decades, numerous studies have reported oxidative stress and inflammation in sepsis patients (21-27). Recent research has indicated that the onset of sepsis leads to increased mitochondrial damage through reactive oxygen species signaling, ultimately resulting in tissue and organ injuries (28-30). However, the distinct mechanisms involved remain unclear (31, 32). Clinical studies have shown that intravenous Dex infusion reduces serum cytokine levels, including IL-1, IL-6, and TNF-α, as well as intra-abdominal pressure, to a greater extent than propofol infusion in patients with severe sepsis after abdominal surgery (3). Chen *et al*. (18) found that Dex treatment significantly reduces pro-inflammatory mediators and oxidative stress markers (MDA and SOD) in the hepatic tissue of an LPS-induced rat sepsis model compared to the control group. Conversely, inhibition of α2-AR by YOH exacerbates LPS-induced injury, underscoring the crucial role of α2-AR in sepsis-induced liver damage. Furthermore, Dex has been shown to protect against systemic inflammation, neuroinflammation, blood-brain barrier disruption, and cognitive dysfunction in CLP-induced septic mice by reducing the levels of IL-1β, IL-6, and TNF-α in the blood and hippocampus (33). Importantly, YOH specifically antagonizes the neuroprotective effects of Dex in the brain. Dex also attenuates CLP-induced acute kidney damage (17), as evidenced by reduced expression of NF-κB and TLR4 in renal tissue, along with decreased inflammatory factors in plasma. Recent studies have demonstrated that Dex has a cardioprotective effect by reducing inflammatory factor expression and preventing ferroptosis, apoptosis, and pyroptosis (34). Collectively, these studies underscore systemic inflammation as the hallmark of sepsis, leading to increased oxidative stress and organ damage. Our study reveals that Dex mitigates ALI by reducing the release of inflammatory factors and MDA while increasing SOD levels, simultaneously, Dex could increase the autophagy manifested by increasing the expression of the protein Beclin1 and decreasing the expression of protein mTOR. These protective effects of Dex are reversed by YOH. Our findings align with previous research and collectively demonstrate the protective effects of Dex against ALI, primarily mediated through α2-AR, and these findings also show that autophagy had close relationships with inflammatory factors and oxidative stress in the protection of Dex on ALI. Further research is needed to explore the relationships among inflammatory factors and oxidative stress, utilizing activators and inhibitors of autophagy to elucidate their roles in ALI.

Apoptosis, also known as type I programmed cell death, is a highly conserved process from *C*.* elegans* to humans. It plays vital roles in vertebrates during development, cellular homeostasis, and tumor suppression. Morphologically, apoptosis is characterized by a signaling cascade that activates executioner caspases, leading to the efficient clearance of intracellular components and eventual phagocytosis of the dying cell. Distinguishing between apoptotic and autophagic cell death is challenging, as these two processes are not mutually exclusive; instead, the type and extent of stress determine whether a cell’s response shifts from adaptive autophagy to apoptotic cell death. Additionally, our data indicate that Dex treatment reduces the levels of pro-apoptotic proteins Bax and cleaved caspase-3 while enhancing the levels of the anti-apoptotic protein Bcl-2 in the lung tissues of septic mice. Bcl-2 is a critical anti-apoptotic protein, while Bax and cleaved caspase-3 are key apoptotic proteins within cells. These findings are consistent with prior evidence suggesting that Dex protects against oxidative stress, inflammation, and apoptosis in sepsis-induced lung injury (35).

As mentioned earlier, sepsis triggers a sudden surge in proinflammatory cytokine production, leading to cell apoptosis and multi-organ damage. Autophagy is initially activated in sepsis and interacts with the inflammatory response to sepsis (36). Pulmonary respiratory failure frequently occurs during the early stages of sepsis, characterized by early decline in autophagy-related mediators alongside increased levels of pro-apoptotic proteins (37). In our study, Dex treatment reversed the increased expression of p-mTOR and reduced Beclin-1 levels induced by sepsis. However, the pro-autophagy effects of Dex were interrupted by YOH treatment, suggesting that Dex’s pro-autophagy effects may also depend on α2-ARs. Beclin1 was initially discovered as a Bcl-2 interacting protein, forming a molecular link between autophagy and apoptosis. The BH3 domain of Beclin1 interacts with Bcl-2, and disrupted interaction between these molecules may lead to dysregulated autophagy and apoptosis. Beclin1-mediated autophagy has been implicated in various inflammatory conditions, with silencing of Beclin1 in human pulmonary artery endothelial cells resulting in reduced expression of proinflammatory genes and mediators that otherwise remain up-regulated in inflammatory diseases. This highlights the role of Beclin1-mediated autophagy in contributing to cytotoxic effects in inflammatory diseases (38). Researchers reported that in LPS-induced heart injury, mTOR was significantly attenuated in Becn1-Tg hearts but was substantially stronger in Becn1^+/-^ mice compared to WT mice. This inverse correlation between mTOR activation and autophagic activity suggests that enhancing Beclin1 signaling can suppress mTOR activation, thereby sustaining autophagy even under severe sepsis conditions, Beclin1 and mTOR had an inseparable relationship in ALI, Beclin1 also acted as the connection of autophagy and apoptosis (39). A study found that after 28 days of CLP-induced ALI in rats, Beclin1 was significantly decreased but treatment of Dex could not rescue the expression of protein Beclin1, this also demonstrated that Beclin1 had a central role in the ALI (40), but in different timepoint of ALI, the effects of Dex may be different. In our study, we confirmed the protection effects of Dex, and we mainly focused on the protein Beclin1 and mTOR in ALI. Future studies should employ conditional knockout mice to elucidate Beclin1’s role in Dex-mediated protection against ALI.

The TGF-β1/Smad signaling pathway, triggered by the cytokine TGF-β1, plays a pivotal role in endothelial barrier function and lung homeostasis. This pathway operates through type I receptor-mediated phosphorylation of Smad 2 and Smad 3. (41). Recent work has demonstrated that down-regulating Smad2/3 activation can modulate the secretion of pro-inflammatory factors, curtail cell death, and alleviate lung inflammation and injury, underscoring its role in maintaining pulmonary integrity and mitigating pulmonary inflammation (41). In our study, the administration of Dex led to a notable decrease in p-Smad2/3 expression within the lung tissue of septic mice. Intriguingly, this protective effect of Dex was significantly dampened in the presence of YOH, implying a plausible association between the beneficial effects of Dex and the p-Smad2/3 signaling pathway (42-44). Previous research has demonstrated that autophagy and the smad2/3 signaling pathway play pivotal roles in regulating inflammatory responses. This study delves into the involvement of autophagy and the smad2/3 signaling pathway as potential mechanisms underlying Dex-mediated treatment for mitigating the inflammatory response in sepsis-induced lung injury. We have observed the activation of autophagy and the smad2/3 signaling pathway in sepsis-induced pulmonary damage. Dex exhibits protective effects against lung injury, manifesting in terms of mitigated pathological changes, reduced levels of inflammatory cytokines, and decreased apoptosis. These protective effects are accompanied by decreased expression of autophagy-related and smad2/3-related proteins in ALI. Notably, YOH treatment significantly diminishes the protective effects of Dex. In conclusion, our results suggest that the reduction in autophagy and smad2/3 signaling pathway activity contributes to the protective action of Dex against ALI. Further research is needed to explore the relationship between autophagy and smad2/3, utilizing activators and inhibitors to elucidate their roles in smad2/3 modulation.

Future investigations should aim to delve deeper into the intricate interplay between Dex, autophagy, and the smad2/3 pathway. Moreover, targeted studies focused on elucidating the specific roles of key molecular targets, such as Beclin1, mTOR, and Smad2/3, may reveal promising avenues for the development of novel therapeutic strategies to combat ALI in septic patients.

## Conclusion

In summary, our study demonstrated that Dex protected against lung injury in a CLP-induced sepsis mouse model. Collectively, our results strongly indicate that autophagy and the smad2/3 signaling pathway actively participate in Dex’s protective mechanisms against ALI. These findings not only enhance our understanding of the complex pathophysiology involved in ALI but also highlight the potential therapeutic utility of Dex in this challenging clinical context.

## Authors’ Contributions

Z L, X Y, and W Y designed the experiments; J X, Y Z, Y W, and R G performed experiments and collected data; C L, J L, and Z L discussed the results and strategy; X Y and W Y supervised, directed, and managed the study; Z L, J L, X Y, and W Y approved the final version to be published

## Data availability

All data are available from the corresponding author upon reasonable request.

## Ethical Approval and Consent to participate

Not applicable.

## Human Ethics

Not applicable.

## Consent for publication

Not applicable.

## Funding

This work was granted by the National Natural Science Foundation of China (No. 81900264), the Shenzhen Science and Technology Innovation Commission (No. JCYJ20210324130609027), the Longgang Medical and Health Science and Technology Project (LGKCYLWS2020040, LGKCYLWS2020044, and LGKCYLWS2020045).

## Conflicts of Interest

The authors declare that they have no competing interests.
